# Modified Sparrow Search Algorithm by Incorporating Multi-Strategy for Solving Mathematical Optimization Problems

**DOI:** 10.3390/biomimetics10050299

**Published:** 2025-05-08

**Authors:** Yunpeng Ma, Wanting Meng, Xiaolu Wang, Peng Gu, Xinxin Zhang

**Affiliations:** 1 School of Information Engineering, Tianjin University of Commerce, Beichen, Tianjin 300134, China; mayunpeng@tjcu.edu.cn (Y.M.);; 2 College of Science, Tianjin University of Commerce, Beichen, Tianjin 300134, China

**Keywords:** modified sparrow search algorithm, swarm intelligence optimization, Latin hypercube sampling, adaptive weighting, Cauchy mutation

## Abstract

The Sparrow Search Algorithm (SSA), proposed by Jiankai Xue in 2020, is a swarm intelligence optimization algorithm that has received extensive attention due to its powerful optimization-seeking ability and rapid convergence. However, similar to other swarm intelligence algorithms, the SSA has the problem of being prone to falling into local optimal solutions during the optimization process, which limits its application effectiveness. To overcome this limitation, this paper proposes a Modified Sparrow Search Algorithm (MSSA), which enhances the algorithm’s performance by integrating three optimization strategies. Specifically, the Latin Hypercube Sampling (LHS) method is employed to achieve a uniform distribution of the initial population, laying a solid foundation for global search. An adaptive weighting mechanism is introduced in the producer update phase to dynamically adjust the search step size, effectively reducing the risk of the algorithm falling into local optima in later iterations. Meanwhile, the cat mapping perturbation and Cauchy mutation operations are integrated to further enhance the algorithm’s global exploration ability and local development efficiency, accelerating the convergence process and improving the quality of the solutions. This study systematically validates the performance of the MSSA through multi-dimensional experiments. The MSSA demonstrates excellent optimization performance on 23 benchmark test functions and the CEC2019 standard test function set. Its application to three practical engineering problems, namely the design of welded beams, reducers, and cantilever beams, successfully verifies the effectiveness of the algorithm in real-world scenarios. By comparing it with deterministic algorithms such as DIRET and BIRMIN, and based on the five-dimensional test functions generated by the GKLS generator, the global optimization ability of the MSSA is thoroughly evaluated. In addition, the successful application of the MSSA to the problem of robot path planning further highlights its application advantages in complex practical scenarios. Experimental results show that, compared with the original SSA, the MSSA has achieved significant improvements in terms of convergence speed, optimization accuracy, and robustness, providing new ideas and methods for the research and practical application of swarm intelligence optimization algorithms.

## 1. Introduction

With the continuous advancement of science and technology, traditional optimization algorithms, such as Newton’s method [1] and gradient descent [2], are increasingly revealing their limitations when facing large-scale, high-dimensional, and nonlinear problems. These methods typically rely on specific assumptions about the problem, making them less adaptable to dynamic optimization scenarios. In contrast, swarm intelligence optimization algorithms [3], due to their simple structure, ease of implementation, and outstanding efficiency and adaptability in solving complex problems, have gradually become essential tools in both research and application. These algorithms simulate the collective behavior of biological populations in nature, relying on cooperation and competition among individuals to search for optimal solutions. They enable effective global search in dynamic and complex environments. Compared to traditional optimization methods, swarm intelligence optimization algorithms offer greater robustness, avoiding the problem of local optima more effectively, thereby enhancing both global search capability and precision.

In the research process of optimization algorithms, many scholars have proposed a series of distinctive optimization methods for different problems. To address the nonlinear optimization challenge in passive positioning using time-frequency differences of moving dual stations, Zhang et al. [4] proposed a hybrid positioning algorithm combining the Cuckoo Search (CS) algorithm and the Newton method. This approach uses the global search results of the Cuckoo Search algorithm as the initial values for the Newton method and iteratively solves for the target position through the Newton method. It effectively overcomes the drawbacks of the slow convergence of the Cuckoo Search algorithm and the sensitivity of the Newton method to the selection of initial values. In the field of optimization algorithms, the projected reflected gradient method is a core approach for solving variational inequalities. The inertial extrapolated projected reflected gradient method proposed in reference [5] optimizes the iterative process by introducing an inertial mechanism, effectively adjusting the search strategy. Experiments show that this method outperforms traditional algorithms in both convergence speed and solution accuracy, providing a new solution for complex optimization problems. Nikita Sakovich et al. proposed a new MAMGD gradient optimization method [6], which utilizes exponential decay, adaptive learning rates, and the discrete second-order derivative of the gradient. Its effectiveness has been verified in scenarios such as the minimization of multivariate real-valued functions and the function approximation of multi-layer neural networks. Experiments indicate that this method features a fast convergence speed, strong stability against fluctuations, and excellent gradient accumulation effects. Li et al. [7] proposed an optimized MTD (Moving Target Detection) algorithm for dynamic target detection based on gradient descent with sampling point weights, optimizing the MTD moving target detection technology. The experimental results show that, compared with traditional methods, this algorithm can more accurately detect the speed of dynamic targets even with a small number of sampling points, significantly improving the accuracy of MTD detection. Ye et al. [8] proposed a multi-objective fuzzy optimization scheduling method for regional power grids based on the distributed Newton method. By iteratively finding the minimum value of the objective function within a given region and transforming multiple objective functions into a single objective function, the computational load of multi-objective scheduling of power grids is greatly reduced. Comparative experiments show that this method significantly improves scheduling efficiency and can effectively meet the economic requirements of power grid operation.

The family of swarm intelligence algorithms is rich and diverse. Common swarm intelligence algorithms include Particle Swarm Optimization (PSO, 1995 [9]), Shuffled Frog Leaping Algorithm (SFLA, 2003 [10]), Artificial Bee Colony Algorithm (ABC, 2005 [11]), Grey Wolf Optimization (GWO, 2014 [12]), Sine Cosine Algorithm (SCA, 2016 [13]), Whale Optimization Algorithm (WOA, 2016 [14]), Harris Hawks Optimization (HHO, 2019 [15]), Chimp Optimization Algorithm (Chimp, 2020 [16]), Sparrow Search Algorithm (SSA, 2020 [17]), Dung Beetle Optimizer (DBO, 2022 [18]), Snow Albation Optimizer (SAO, 2023 [19]), Chinese Pangolin Optimizer (CPO, 2025 [20]), and Mirage Search Optimization (MSO, 2025 [21]). These algorithms are inspired by the collective behaviors of various natural organisms, such as the foraging of birds, the food-seeking behavior of bees, and the hunting strategies of whales. They employ a hybrid search strategy that combines local exploration and global exploitation. These algorithms have found widespread applications in diverse fields.

In successive iterations, population diversity in population-based optimization algorithms often decreases, leading to premature convergence to local optima. To address this, various advanced learning strategies have been proposed. Elsisi et al. [22] introduced an Improved Gray Wolf Optimization (IGWO) algorithm that enhances the exploration–exploitation balance by incorporating a novel learning mechanism and a Fitness–Distance Balancing (FDB) technique, improving global search and reducing the risk of local optima without additional parameters. Chen et al. [23] enhanced the Whale Optimization Algorithm (WOA) by adding a nonlinear convergence factor to better balance exploration and exploitation, and also introduced an adaptive weighting strategy and a stochastic differential change strategy to speed up convergence and prevent premature trapping in local optima, with successful application to signal denoising. Liu et al. [24] improved the Chimpanzee Optimization Algorithm (ChOA) with adaptive inertia weights and a flight strategy, and later developed the IChOA-KFC algorithm for RGB-D image segmentation, demonstrating significant performance gains. Zhang et al. [25] proposed an Improved Sine Cosine Algorithm (ISCA) to enhance population diversity and balance exploration and exploitation through uniform initialization and nonlinear strategies, successfully optimizing BiLSTM model parameters to improve prediction accuracy. Javaheri et al. [26] developed a discrete and dyadic-based version of the Harris Hawk Optimization (HHO) algorithm, incorporating eight dyadic learning strategies to improve convergence rates and applied it effectively to job scheduling for computational providers, showing notable improvements in optimization efficiency and effectiveness.

The Sparrow Search Algorithm (SSA) was proposed by Professors Xie and Shen from Donghua University, inspired by the natural patterns of sparrow foraging and defense behaviors. The algorithm exhibits fast convergence speed, high precision, and strong robustness, demonstrating excellent performance in solving complex optimization problems. However, when handling certain complex problems, SSA may still fall into local optima, and its performance is highly sensitive to parameter selection. The overly rapid convergence speed can sometimes lead to a trade-off between accuracy and efficiency.

Recent advancements in the Sparrow Search Algorithm (SSA) have led to its widespread application across various fields, including UAV trajectory planning, multi-threshold image segmentation, wireless sensor network coverage, job shop scheduling, and price trend prediction [27,28,29]. However, SSA faces challenges in the later stages of iteration due to its direct jump-based update mechanism. This can result in a decline in population diversity and reduced search efficiency. As a result, the algorithm tends to converge slowly and often gets trapped in local optima. To address these issues, researchers worldwide have proposed several enhanced versions of SSA. For instance, Zhou et al. [30] proposed a multi-strategy improved SSA (RSSA), which integrates coarse-to-fine data reasoning and introduces low-differential sequences for population initialization, thereby enhancing the global search capability of the algorithm. Another study [31] introduced an improved SSA based on adaptive t-distribution and golden sine functions. This method uses an adaptive t-distribution mutation to perturb individual positions, helping the algorithm escape from local optima. A further enhancement, the Adaptive Spiral Flight Improved Sparrow Search Algorithm (ASFSSA) [32], incorporates self-adaptive spiral flight patterns to boost the algorithm’s search performance. Additionally, an SSA algorithm based on Sobol sequences was proposed [33], combining longitudinal and lateral crossover strategies to improve search efficiency. Another variant, the Chaos Sparrow Search Algorithm (CSSA) [34], was applied to construct an adaptive network model (CSSA-SCN) for addressing challenges in large-scale data regression and classification. Furthermore, an Adaptive Sparrow Search Algorithm (ASSA) [35] was employed for optimal model parameter identification in proton exchange membrane fuel cells (PEMFCs), demonstrating its effectiveness in PEMFC stack optimization in several case studies. Finally, the improved SSA (CASSA) [36] was designed specifically for 3D path planning of UAVs, with a focus on optimizing the path while minimizing collision risks. Enhanced SSA algorithms have also been integrated into frameworks for short-term power load forecasting [37], where they help optimize the parameters of gated recurrent unit neural networks to improve forecasting accuracy.

According to the “No Free Lunch” (NFL) theorem [38], the most effective optimization strategy is problem-dependent, and no single algorithm can consistently outperform others across all problem landscapes. Therefore, optimization strategies must be adapted and improved according to the specific characteristics of the problem at hand. While several modified versions of the Sparrow Search Algorithm (SSA) have been proposed—such as those that adjust the algorithm’s structure or incorporate new operations to enhance global search capability—these approaches still exhibit certain limitations when faced with more complex or dynamic optimization problems. Consequently, there is an urgent need to further optimize the SSA to overcome the shortcomings of existing modifications and improve its performance across a wider range of complex problems. This paper proposes a modified version of the Sparrow Search Algorithm (MSSA), featuring three critical improvements. First, Latin Hypercube Sampling (LHS) [39] is utilized to augment population diversity, thereby enhancing the global search capability and accelerating convergence toward the global optimum. Second, an adaptive weighting mechanism is incorporated in the position update process for the producers, ensuring balanced exploration in the early iterations and reducing the likelihood of premature convergence in later stages. Finally, the algorithm is further strengthened by integrating Cauchy mutation and Cat perturbation techniques, which effectively disrupt the search process and enable the algorithm to escape local optima.

The primary contributions of this paper are briefly summarized as follows:

(1) A new optimization algorithm MSSA is proposed. It enhances population diversity with Latin Hypercube Sampling (LHS) during initialization, enhances search efficiency through an adaptive weighting mechanism in the discovery phase, and strengthens global search with Cauchy mutation and Cat perturbation strategies.
(2) Based on the tests conducted on 23 benchmark functions, CEC2019 test functions, and three engineering optimization problems, the MSSA was also compared with the deterministic algorithms DIRECT and BRIMIN on 100 five-dimensional GKLS test functions to verify its global optimization ability.
(3) The algorithm’s effectiveness is verified through statistical analysis of mean and standard deviation. The Wilcoxon’s rank-sum test at a 0.05 significance level shows a significant difference.
(4) The modified MSSA is applied to a 20 × 20 robot path planning problem, validating its performance in dynamic obstacle avoidance and path optimization, providing strong algorithmic support for practical applications.

The structure of this paper is arranged as follows: Section 2 reviews the principles and development history of the basic Sparrow Search Algorithm; Section 3 elaborates on the implementation process of the Modified Sparrow Search Algorithm (MSSA) in detail. In Section 4, the optimization performance of the MSSA is compared with that of other swarm intelligence algorithms on 23 benchmark test functions and CEC2019 test functions. Its global optimization ability, convergence speed, and algorithm stability are evaluated, and the statistical significance is assessed through the Wilcoxon test. Meanwhile, the MSSA is compared with deterministic algorithms on 100 five-dimensional GKLS test functions to verify its global optimization ability. Section 5 validates the effectiveness of the MSSA in handling complex constraints through three engineering design problems, highlighting its advantages. Section 6 demonstrates the effectiveness of the MSSA in industrial applications by taking the robot path planning problem as an example. Finally, Section 7 summarizes the main research content and proposes potential directions for future research.

## 2. Sparrow Search Algorithm (SSA)

In the SSA algorithm, the sparrow population is divided into three types: producers, scroungers, and scouters. Producers, with higher energy, lead the population in exploring food sources; scroungers follow producers and compete for resources; scouters stay alert and guide the population to safety upon detecting threats. The SSA generates an initial population of N sparrow individuals in a D-dimensional space through random initialization.

### 2.1. Producer Position Updates Phase

During each iteration, the position update formula for the producers is described by Equation (1):

(1) $$x_{i,j}^{t+1}=\left\{\begin{array}{c} x_{i,j}^{t}\cdot \exp\left(\frac{-i}{\alpha \cdot T}\right),\;\;\;\;R_{2}<ST\\ \;x_{i,j}^{t}+Q\cdot L,\;\;\;\;\;\;\;{\;\;\;R}_{2}\geq ST \end{array}\right.$$

Here, t represents the current iteration number, and j=1,2,3,...,D. T is a constant representing the maximum number of iterations. $x_{i,J}^{t+1}$ denotes the position of the i sparrow in the j dimension, α∈(0,1] is a random number. Both ST(ST∈[0.5,1]) and $\left(R_{2}\in [0,1]\right)$ represent the warning value and safety value, respectively. Q is a random number following a normal distribution. L is a 1×D matrix with all elements 1.

### 2.2. Scrounger Position Updates Phase

The position update formula for the scroungers is described by Equation (2):

(2) $$x_{i,j}^{t+1}=\left\{\begin{array}{c} Q\cdot \exp\left(\frac{x_{worst}^{t}-x_{i,j}^{t}}{i^{2}}\right)\;\;\;\;\;\;\;\;\;\;\;\;\;\;\;\;\;\;\;\;\;\;if\;\;\;\;i>\frac{n}{2}\;\\ x_{p}^{t+1}+\left|x_{i,}^{t}-x_{p}^{t+1}\right|\cdot A^{+}\cdot L\;\;\;\;\;\;\;\;\;otherwise\;\;\;\; \end{array}\right.$$

where $x_{p}^{t+1}$ is the location with the best food resource in the t+1 iteration, $x_{worst}^{t}$ is the location with the location with the worst resource, A is a 1×d matrix with elements randomly replicated as 1 or −1, $A^{+}=A^{T}{\left(AA^{T}\right)}^{-1}$.

### 2.3. Scouter Position Updates Phase

The position update formula for the scouters is described by Equation (3):

(3) $$x_{i,j}^{t+1}=\left\{\begin{array}{c} x_{best}^{t}+\beta \cdot \left|x_{i,j}^{t}-x_{best}^{t}\right|\;\;\;\;\;\;\;\;\;if\;\;\;f_{i}>f_{g}\;\\ x_{i,j}^{t}+K\left(\frac{\left|x_{i,j}^{t}-x_{worst}^{t}\right|}{(f_{i}-f_{w})+\varepsilon }\right)\;\;\;\;\;\;\;\;\;else\;\;f_{i}<f_{g} \end{array}\right.$$

where $x_{best}^{t}$ is the current global best position. β obeys a standard normal distribution, K(K∈[−1,1]) is a random number. $f_{i}$ is the fitness value of the current sparrow individual. $f_{g}$ and $f_{w}$ are the optimal and worst fitness values in the current iteration, respectively. ε is the smallest constant.

## 3. A Modified Sparrow Search Algorithm

Although the SSA offers advantages such as ease of implementation, fast convergence speed, and strong robustness, it still faces challenges such as a tendency to get trapped in local optima and insufficient convergence accuracy. In order to overcome the shortcomings of the SSA, this paper proposes a modified sparrow search algorithm (MSSA). The improvement strategy of the MSSA focuses on three main areas. Firstly, LHS is employed to enhance population diversity, this approach helps prevent premature convergence to local optima and enhances the algorithm’s ability to escape local traps, thereby strengthening its global search performance. Secondly, an adaptive weighting mechanism is incorporated into the position update process of the producers. As the algorithm progresses, the exploration weight is gradually reduced, shifting the focus towards a more refined exploitation of the information gathered. This adaptive strategy mitigates the risk of premature convergence, enabling the algorithm to effectively exploit promising regions of the search space in the later stages of the optimization process. Finally, the algorithm is further enhanced by integrating Cauchy mutation and Cat perturbation techniques. Cauchy mutation introduces heavy-tailed perturbations to the search process, allowing the algorithm to escape local optima by exploring more diverse regions of the search space. Additionally, the Cat perturbation simulates random walk behaviors, enabling large-scale search disruptions that help avoid stagnation in suboptimal solutions. The detailed improvement strategy is summarized as follows.

### 3.1. Latin Hypercube Sampling

Latin Hypercube Sampling (LHS), proposed by McKay et al., is a multi-dimensional stratified sampling technique that efficiently samples within the distribution intervals of variables. The basic principle involves dividing the interval [0,1] into N equal subintervals and performing independent random sampling with equal probability within each subinterval, ensuring that the samples are evenly distributed across the entire interval.

The initial population is generated in a D-dimensional space with a population size of N. By incorporating Latin Hypercube Sampling (LHS), the population initialization strategy for MSSA is developed. The specific process is as follows:

(1) First, determine the population size N and the dimensionality D of the population.
(2) Define the range of variables x as [ub,lb], where ub and lb are the upper and lower bounds, respectively.
(3) Divide the range [ub,lb] of each variable x into N equal intervals. The width of each sub-interval is Δx=(ub−lb)/N.
(4) Randomly select a point from each interval in every dimension. A random number generator within the range of [0, 1] can be used in each sub-interval.
(5) Combine the points selected in all dimensions to form the initial population. After sampling one point in each sub-interval of all D dimensions, an individual in the population is formed. Repeat this process N times to obtain the initial population of the MSSA algorithm.

**Figure 1:**
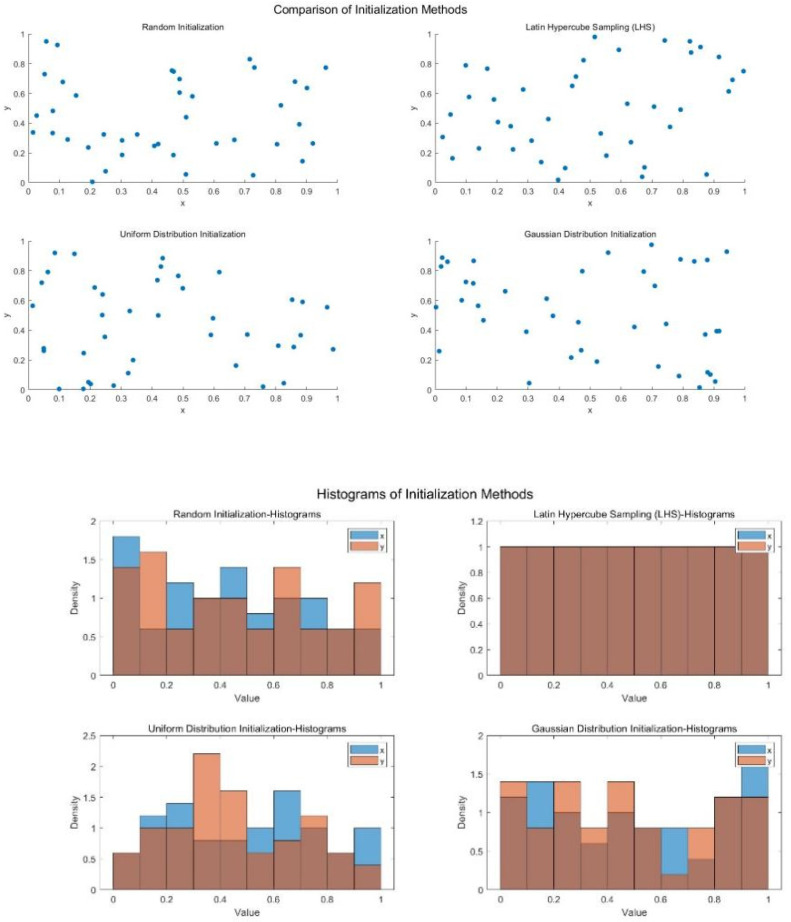
Comparison of sampling distributions (N=40).

**Figure 2:**
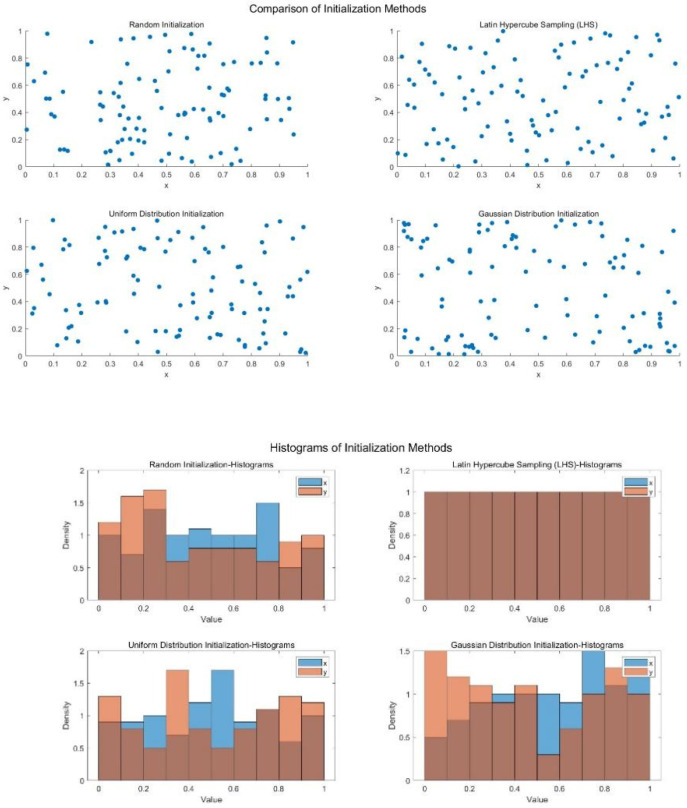
Comparison of sampling distributions (N=100).

Table 1 summarizes four common sampling methods along with their characteristics, advantages, and disadvantages, and compares LHS with these methods. By drawing 40 and 100 points in the interval [0, 1], the scatter plots in Figure 1 and Figure 2 clearly demonstrate the significant advantages of the LHS sampling method, which effectively distributes the samples uniformly across the entire interval.

### 3.2. Adaptive Weighting Mechanism

The adaptive mechanism dynamically adjusts the algorithm’s position based on the current search state, improving its adaptability and robustness. It guides the algorithm towards the optimal solution, speeding up convergence and helping escape local optima. In the sparrow population finder position update stage, when R2<ST, the original update method with the number of iterations increases, the population diversity decreases sharply, to address this issue, an adaptive mechanism is introduced to ensure the algorithm continues broad searching in later iterations. The update strategy is as follows:

(4) $$X_{i,d}^{t+1}=X_{i,d}^{t+1}\cdot w_{1}$$

(5) $$w_{1}=l_{1}\cdot \exp(-\frac{r\cdot i}{\alpha \cdot T})+\frac{2l_{2}}{\pi }abs[\arctan(\frac{1+r\cdot i}{\alpha^{2}\cdot T})]$$

where $l_{1}\in (0,1)$, and $l_{1}+l_{2}=1$, *r* is a constant greater than 1, α is a random number within (0, 1) and $w_{1}$ is an adaptive weight. Here take $l_{1}=0.4$, r=2.

The weight parameter $w_{1}$ is designed to dynamically balance exploration and exploitation through two components: (1) an exponential decay term $l_{1}\cdot \exp(-\frac{r\cdot i}{\alpha \cdot T})$, where the decay rate reduces global exploration reliance as iterations i progress, and (2) a compensatory arctangent term $\frac{2l_{2}}{\pi }abs[\arctan(\frac{1+r\cdot i}{\alpha^{2}\cdot T})]$, scaled to the interval $\left(0,l_{2}\right)$, which grows monotonically with i to sustain late-stage exploration while permitting localized exploitation. This dual-term structure ensures progressive focus refinement without premature convergence, critical for navigating complex search spaces.

**Figure 3:**
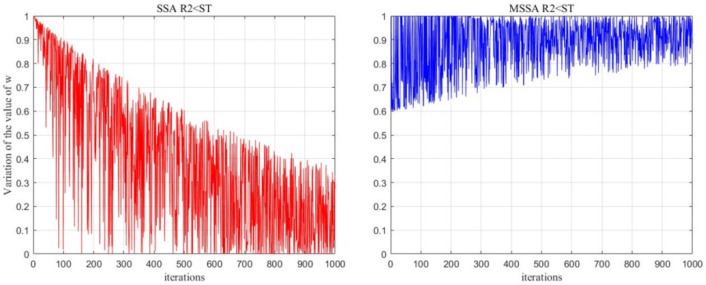
Trajectory diagram of w and $w_{1}$.

The maximum number of iterations is set to 1000 in order to verify the effectiveness of the proposed adaptive weights $w_{1}$. As shown in Figure 3, in the case of R2<ST, it can be observed that the raw SSA $w=\exp\left(\frac{-i}{\alpha \cdot T}\right)$ decreases rapidly with the increase in the number of iterations up to 0.3. This indicates a sharp decline in the diversity of the original SSA population in later iterations, hindering the balance between global exploration and local search. However, the improved $w_{1}$ solves this problem well.

### 3.3. Cauchy Mutation and Cat Disturbance Strategy

In order to solve the problem that the sparrow search algorithm is easy to fall into the local optimum, this paper adopts the Cauchy variation [40] and Cat perturbation strategy [41] in order to increase the diversity of the population, so as to improve the global search ability of the algorithm. The Cauchy variation uses its long-tailed distributional properties to introduce a larger perturbation around the currently mutated sparrow individual, which helps to jump out of the locally optimal solution. Cat mapping is a two-dimensional invertible chaotic mapping with good traversal uniformity, and the generated chaotic sequences are uniformly distributed in [0, 1], which effectively improves the diversity of the population and the global search ability.

After each iteration, the $f_{m}$ of the sparrow population is calculated by Equation (6). If the $f_{i}<f_{m}$, the Cauchy variation is used to introduce the perturbation, see Equation (7); otherwise, the Cat perturbation is used, see Equation (8). In the Cauchy variation strategy, new solutions are generated by applying the Cauchy variation operator to perform a variation operation at the optimal solution location. And, in Cat perturbation, its chaotic properties are utilized to obtain new population solutions. This design can more effectively control the triggering conditions of the perturbation strategy and flexibly adjust the perturbation according to the performance of the population, thus improving the global search capability of the algorithm.

(6) $$f_{m}=\frac{1}{N}\sum_{N}^{i=1}f_{i}$$

(7) $$x_{newbest}=x_{best}+x_{best}\times Cauchy(0,1)$$

(8) $$\left[\begin{array}{c} x_{N+1}\\ y_{N+1} \end{array}\right]=\left[\begin{array}{c} 1\;\;\;1\\ 1\;\;\;2 \end{array}\right]\cdot \left[\begin{array}{c} x_{N}\\ y_{N} \end{array}\right]\mathrm{mod}1=c\left[\begin{array}{c} x_{N}\\ y_{N} \end{array}\right]\mathrm{mod}1$$

Cauchy(0,1) in Equation (7) is the standard Cauchy distribution. Equation (8) is a two-dimensional cat mapping equation where $x\;\mathrm{mod}1=x-[x],C=\left[\begin{array}{c} 1\;\;\;1\\ 1\;\;\;2 \end{array}\right]$.

For the standard Cauchy distribution Cauchy(0,1), its probability density function is $f(x)=1/(\pi (1+x^{2}))$. When generating a new solution using Equation (7), due to the long-tail property of the Cauchy distribution, a Cauchy random variable can take values far from zero with a non-negligible probability. For example, if $x_{best}=5$ and a Cauchy random variable z=3 is sampled from Cauchy(0,1), then $x_{newbest}=5+5\times 3=20$. Such a large perturbation has the potential to move the solution to a new region of the search space, enabling the algorithm to escape from local optimal solutions.

Regarding the cat perturbation, assume that it starts from the initial point $\left[\begin{array}{c} x_{0}\\ y_{0} \end{array}\right]=\left[\begin{array}{c} 0.2\\ 0.3 \end{array}\right]$. Using Equation (8), $\left[\begin{array}{c} x_{1}\\ y_{1} \end{array}\right]=\left[\begin{array}{c} 1\;\;\;1\\ 1\;\;\;2 \end{array}\right]\cdot \left[\begin{array}{c} 0.2\\ 0.3 \end{array}\right]=\left[\begin{array}{c} \;\;\;0.2+0.3\\ 0.2+2\times 0.3 \end{array}\right]=\left[\begin{array}{c} 0.5\\ 0.8 \end{array}\right]$. After performing the mod1 operation, this situation remains unchanged. If further iteration is carried out $\left[\begin{array}{c} x_{2}\\ y_{2} \end{array}\right]=\left[\begin{array}{c} 1\;\;\;1\\ 1\;\;\;2 \end{array}\right]\cdot \left[\begin{array}{c} 0.5\\ 0.8 \end{array}\right]=\left[\begin{array}{c} \;\;\;0.5+0.8\\ 0.5+2\times 0.8 \end{array}\right]=\left[\begin{array}{c} 1.3\\ 2.1 \end{array}\right]$, and after the mod1 operation,$\left[\begin{array}{c} x_{2}\\ y_{2} \end{array}\right]=\left[\begin{array}{c} 0.3\\ 0.1 \end{array}\right]$. This process generates a chaotic sequence of points, which can be used to perturb the population and increase its diversity.

**Figure 4:**
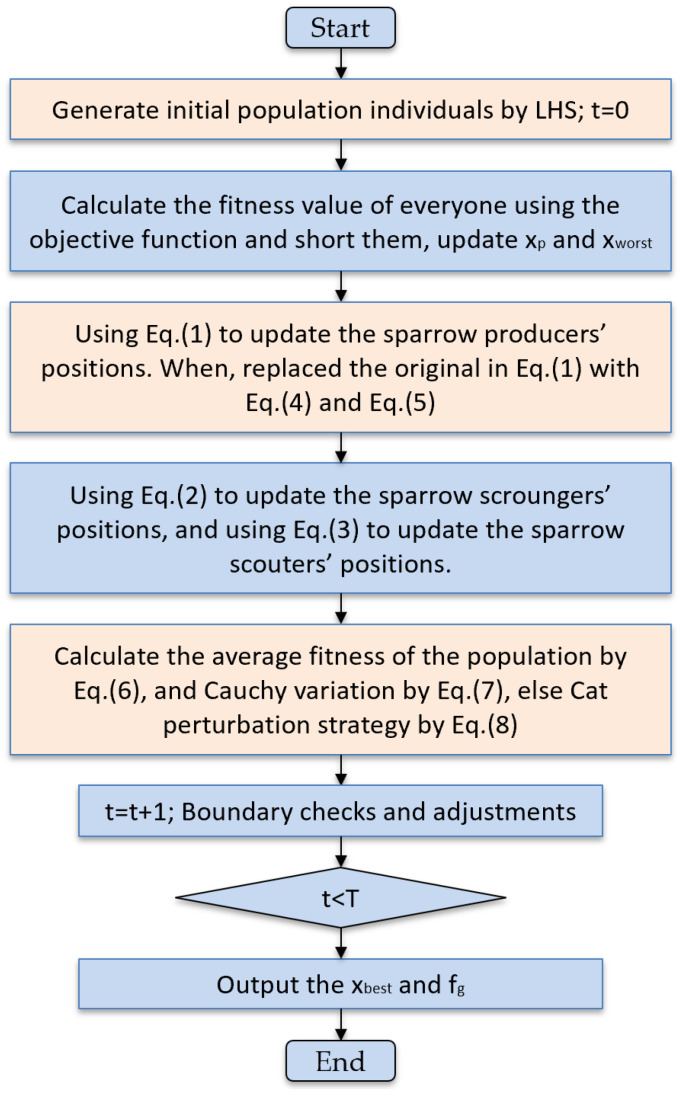
Flowchart of MSSA.

The pseudo-code for the MSSA is given in Algorithm 1, and the overall flowchart of the algorithm is given in Figure 4.
**Algorithm 1:** Pseudo-code of MSSA**Input:** N, D, T, PD, SD, $R_{2}$, ST Initialized population individuals $x_{i}(i=1,2,...,N)$ generated Latin hypercube sampling (LHS) within the D−dimensional problem space.**Output:** $x_{best}$, $f_{g}$ 1: **While** t<T do 2:    Rank the fitness values, identify the current best individual $f_{g}$ and worst individuals $f_{w}$, 3:   **for** i=1:PD 4:      Using Equation (1) to update the sparrow producers’ positions. When R2<ST, replaced the original in Equation (1) with Equation (4) and Equation (5). 5:   **end for** 6:   **for**
i=(PD+1):N 7:   Using Equation (2) to update the sparrow scroungers’ positions. 8:   **for** i=1:SD 9:    Using Equation (3) to update the sparrow producers’ positions. 10:    **end for** 11:    Calculating the average fitness of the population $f_{m}$ by Equation (6). 12:    **if**
$f_{i}<f_{m}$ 13:    Cauchy variation by Equation (7) was employed to perturb sparrow populations in instances where the fitness of individual sparrows fell below the average fitness. 14:    **else** 15:    Sparrow populations were perturbed utilizing the Cat perturbation strategy by Equation (8). 16:    Boundary checks and adjustments 17:    Obtain the current new location; 18:    If the current new location is superior to the previous one, update it; 19:    t=t+1 20: **end while** 21: **return** $x_{best}$, $f_{g}$.

### 3.4. Complexity Analysis

In this subsection, the selection of time complexity and space complexity as evaluation metrics offers a comprehensive assessment of an algorithm’s efficiency and feasibility. Time complexity influences the execution speed, particularly when handling large-scale datasets, while space complexity quantifies memory usage, which affects performance in memory-constrained environments. Evaluating these two dimensions ensures the operability and scalability of MSSA under varying conditions.

The space complexity of the algorithm is determined using the Big O notation. For the standard SSA, the space complexity is O(N×D), where N represents population size, D represents dimension. According to the flowchart and pseudo-code of MSSA, the introduction of an adaptive inertia coefficient leads to the addition of a constant scalar, which does not significantly affect the space complexity and can be considered negligible. Additionally, MSSA requires the storage of each individual’s historical best solution, which results in a space requirement of O(N×D). Furthermore, MSSA must store the global best solution, contributing a space complexity of O(D). Overall, the total space complexity of MSSA remains O(N×D), which is the same as that of the SSA algorithm. Thus, although MSSA incorporates performance improvements, its space overhead remains equivalent to that of the SSA algorithm, maintaining the same level of space efficiency.

The time complexity of the algorithm is determined using the big O notation. In SSA, the initialization phase is specifically formulated to generate a set of prospective solutions intended for subsequent exploration and optimization. The process encompasses the generation of initial solutions, determination of parameter settings, and execution of other essential operations, with a computational complexity of O(N). At this juncture, the evaluation of adaptation becomes imperative to thoroughly appraise the efficacy and caliber of prospective solutions. Overall, the time complexity of the SSA algorithm can be represented as $O\left(T\times N\right)+O(T\times N\times D)$, where T represents the maximum number of iterations. The difficulty in updating the Sparrow Search Algorithm is determined by the neighborhood search difficulty and the employed update strategy. Consequently, the runtime complexity of the SSA is $O\left(N\times (T+T\times D+1)\right)$. In MSSA, the runtime complexity remains unaltered at $O\left(N\times (T+T\times D+1)\right)$.

## 4. Performance Testing of Functions

To evaluate the effectiveness of the proposed Modified Sparrow Search Algorithm (MSSA), a performance test was conducted using 23 benchmark functions [42] from multiple dimensions, including global search ability, local exploitation ability, and optimization-seeking capability. Specifically, unimodal functions F1–F7, each with a single global extremum, were employed to examine the local exploitation ability of MSSA. Multimodal functions F8–F13, which possess multiple local extrema, were utilized to test the algorithm’s robustness in handling multiple optimal solutions. Additionally, fixed-dimensional multimodal functions F14–F23 were applied to assess the performance of MSSA in low-dimensional spaces. Furthermore, 10 CEC2019 benchmark functions [43] were introduced to further validate the algorithm’s performance. 

The experiments were carried out on a computer equipped with a Windows 10 64-bit operating system, an Intel Core i5 processor (2.60 GHz), and 8 GB of RAM, using MATLAB R2022b for simulation. The population size was set to 40, and the maximum number of iterations was configured as 500. The parameter settings of MSSA were consistent with those of the original Sparrow Search Algorithm (SSA), while the other compared algorithms adopted their default parameter configurations. The parameter settings are shown in Table 2. The convergence accuracy, stability, convergence speed, and overall performance advantages of the algorithms were comprehensively evaluated by calculating the mean and standard deviation based on 20 independent runs. To objectively evaluate the performance differences between the MSSA and other comparative algorithms, we conducted a statistical analysis based on the rank-sum test at a significance level of α=0.05. For each test task, we computed the rank sums of MSSA and each competing algorithm. At the 0.05 significance level, we compared the calculated rank sums against standard critical values. If the obtained rank sum fell outside the critical range, we rejected the null hypothesis, concluding that the performance difference between MSSA and the competing algorithm was statistically significant. Otherwise, we failed to reject the null hypothesis, indicating no significant performance difference. As a non-parametric test, the rank-sum test does not assume any specific data distribution, making it robust for analyzing diverse performance metrics in complex testing scenarios.

### 4.1. Performance Testing on 23 Benchmark Functions

**Figure 5:**
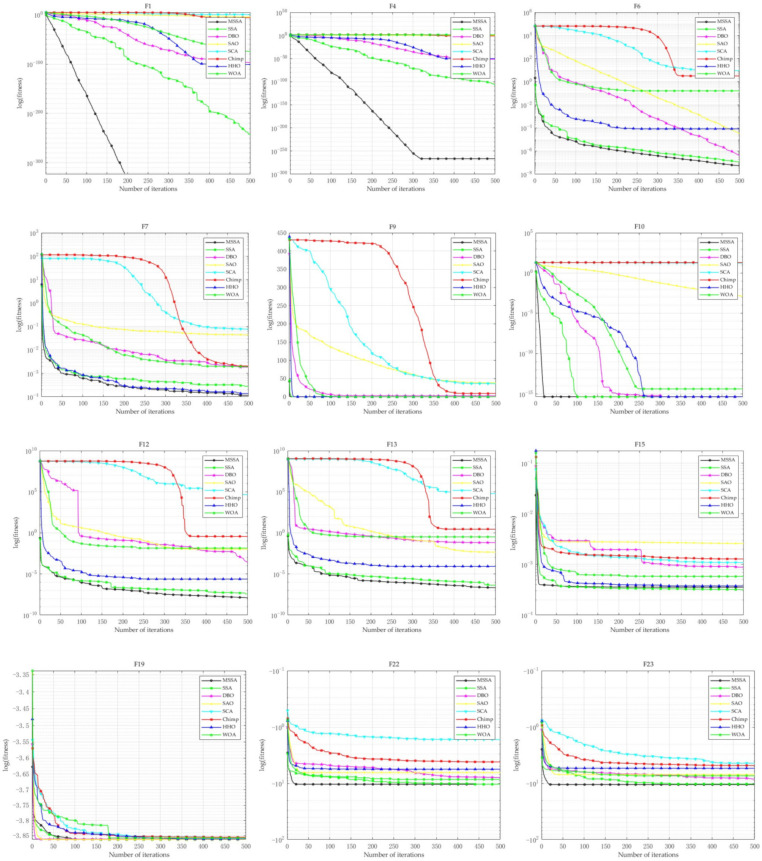
Comparison of convergence curves of MSSA and other algorithms on 23 benchmark functions.

In this subsection, the performance of MSSA and other algorithms is evaluated using 23 benchmark functions, each with 30 dimensions (though they can also be configured with 2, 10, 50, or 100 dimensions). The comparison focuses on their exploitation ability, exploration ability, and convergence capability. For each test function, the MSSA and other algorithms were independently run 20 times to identify the global optimal solution. The mean and standard deviation of the results from these 20 trials were then computed, with the mean representing the algorithm’s convergence accuracy and the standard deviation reflecting its stability. The experimental results are shown in Table 3. The comparison of the convergence curves of MSSA and other algorithms across 23 benchmark functions is shown in Figure 5.

The experimental results presented in Table 3 clearly demonstrate that the improved MSSA algorithm performs excellently on the benchmark functions F1 through F7. Not only does it achieve optimal average values, but it also exhibits remarkable stability in terms of variance, indicating its strong robustness. For the functions in the range of F8 to F13, although MSSA performs slightly worse than the HHO and WOA algorithms on F8, its optimization results on the other functions remain superior to those of the comparison algorithms, highlighting its outstanding performance in most cases. In the more complex function set from F14 to F23, MSSA continues to exhibit strong competitiveness, with optimization results for most benchmark functions being highly satisfactory. However, it falls short of reaching the optimal solution for functions F15, F19, and F20, showing a slight performance degradation in these specific cases. Additionally, the Wilcoxon rank-sum test further verifies the statistical independence of the MSSA algorithm in terms of optimization performance. In the Wilcoxon rank-sum test, a significance level of 0.05 is conventionally selected as it represents a 5% probability of erroneously rejecting the null hypothesis (i.e., a Type I error) when the null hypothesis is actually true. This threshold provides a balance between the test’s sensitivity and specificity, thereby reducing the risk of overlooking meaningful differences due to an excessively stringent significance level. When the *p*-value is close to 0.05, the results should be interpreted with caution, as they lie on the margin of statistical significance. In such cases, it is advisable to further validate the findings through studies with larger sample sizes to enhance the robustness and reliability of the conclusions. The results indicate significant differences between the MSSA and the comparison algorithms, with MSSA outperforming them in terms of statistical significance. As shown in Figure 5, the MSSA possesses a significant convergence speed advantage, rapidly approaching the global optimum, demonstrating both its efficiency and practicality. While there are areas that could be further refined, it remains a viable and efficient optimization algorithm overall.

**Figure 6:**
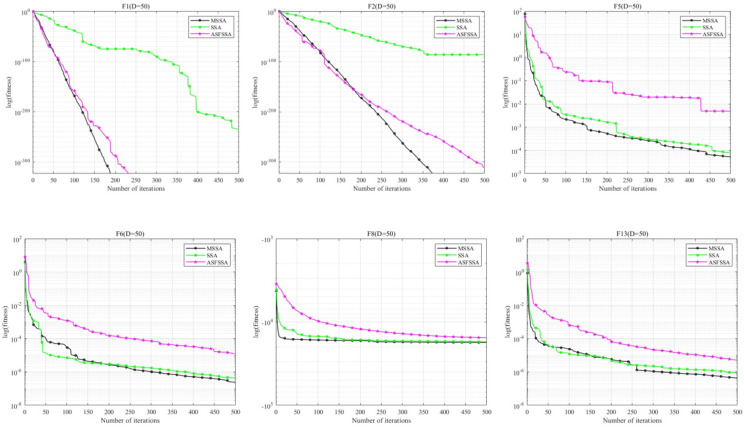

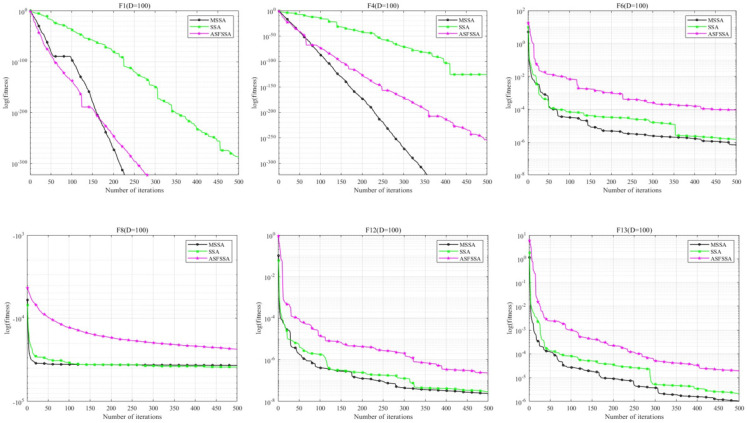
Comparison of convergence curves of MSSA and other algorithms on F1−F13.

To further evaluate the performance of the improved MSSA, we selected the F1–F13 benchmark functions and conducted experiments in higher-dimensional spaces (D=50 and D=100). The MSSA was compared with the original SSA and the ASFSSA, with the parameter settings consistent with those described earlier. As shown in Table 4 and Figure 6, MSSA achieves the optimal solution for the majority of test functions. Moreover, it exhibits a significantly faster convergence rate compared to both the original SSA and ASFSSA, which effectively validates the effectiveness and superiority of the proposed improvements in MSSA.

**Figure 7:**
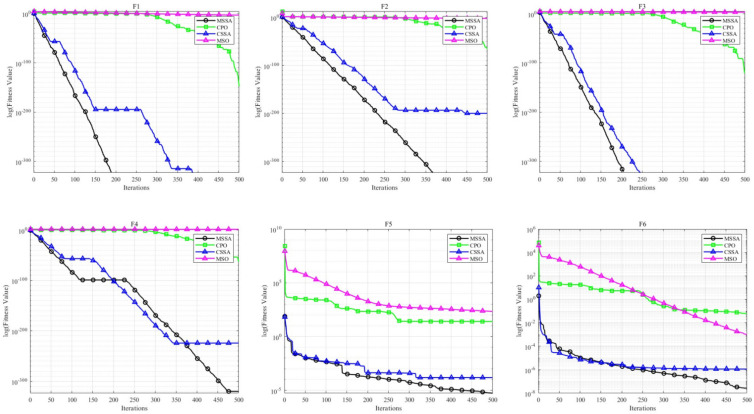

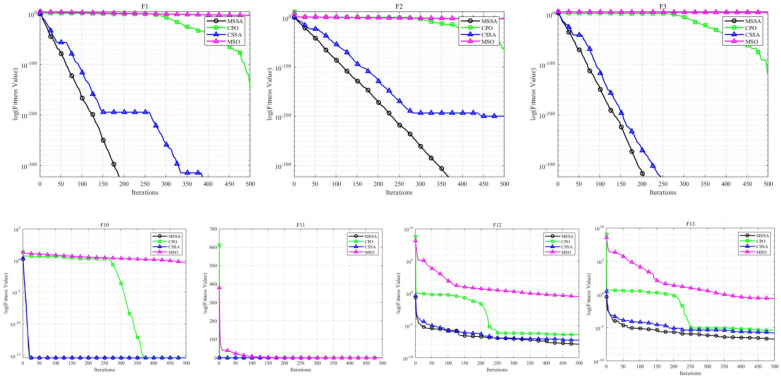
Comparison of convergence curves of MSSA and other algorithms on F1−F13 (D=30).

To further assess the performance of MSSA, its discoverer proportion was adjusted from 0.5 to 0.8. The adjusted MSSA was then compared with the Chinese Pangolin Optimizer (CPO) and Mirage Search Optimization (MSO) algorithms. Using F1–F13 benchmark functions in a 30-dimensional space, this study aimed to verify the performance enhancement of MSSA under alternative parameter configurations and its competitiveness against recent algorithms. As presented in Table 5 and Figure 7, MSSA outperforms MSO and CPO across various settings, validating its stability and effectiveness.

### 4.2. Performance Testing on CEC2019

**Figure 8:**
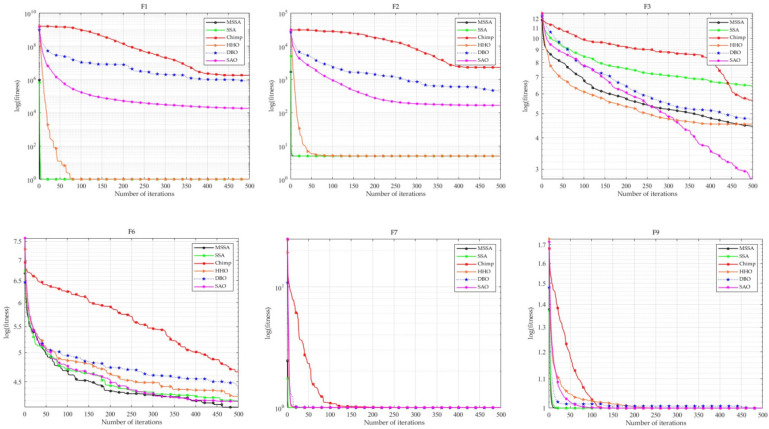
Comparison of convergence curves of MSSA and other algorithms on CEC2019.

The CEC2019 test functions consist of 10 test functions, denoted as F1 to F10, as detailed in Table 6 Due to the stochastic nature of the algorithms, all conclusions are derived from the results of 20 independent runs, which include the average values, standard deviations, and Friedman test statistics for each iteration. MSSA was compared with five other algorithms on the CEC2019 test functions set to further validate the effectiveness of its improvements. The parameter settings are provided in Table 2. The experimental results are provided in Table 6 (For the CEC2019 test results, the evaluation is based on values rounded to four decimal places). The comparison of convergence curves of MSSA and other algorithms are provided in Figure 8.

From Table 6 and Figure 8, it is evident that MSSA exhibits a faster convergence rate. Except for its slightly lower performance on F3 compared to SAO, MSSA outperforms all other algorithms on the remaining functions. The average rank results from the Friedman test further confirm that MSSA achieves the best overall performance. These findings highlight the superior effectiveness of MSSA on the CEC2019 test functions.

### 4.3. Performance Testing on GKLS Functions

When evaluating the global optimization ability of algorithms, deterministic algorithms and heuristic algorithms belong to different technical categories [44]. Deterministic algorithms are based on rigorous mathematical theories and rule systems. Algorithms such as DIRECT [45] and BIRMIN [46] employ systematic search strategies, and their operation processes follow strict logic, exhibiting a high degree of predictability and certainty. Taking the DIRECT algorithm as an example, it demonstrates excellent exploration capabilities in low-dimensional spaces, but its performance significantly deteriorates in high-dimensional space scenarios. In contrast, heuristic algorithms often simulate natural phenomena or draw on human experience and adopt a random exploration approach to search for approximate optimal solutions. To comprehensively and systematically study the global optimization performance of the Modified Sparrow Search Algorithm (MSSA), this study constructs 100 five-dimensional simple test functions with the aid of the GKLS generator [47]. By constructing an operation area and connecting deterministic algorithms and heuristic algorithms through visualization means [48], an effort is made to break down the barriers between theory and practice, and thus conduct an in-depth analysis of the characteristics and effectiveness of these two types of algorithms.

In this study, the objective of the algorithm is to find a solution within the given number of iterations, where the error between this solution and the global optimal value is less than the preset tolerance ($\varepsilon =10^{-4}$). Once this condition is met, the corresponding problem is considered to have been successfully solved.

In order to intuitively present the operation characteristics of the DIRECT and BIRMIN algorithms for simple and difficult five-dimensional problems, this paper plots a line chart showing the relationship between the number of function evaluations and the number of successfully solved problems. Among them, the horizontal axis represents the number of function evaluations, which is displayed at intervals of 1000 function evaluations. If there is a case of a successful solution, the range of the horizontal axis is from the minimum value to the maximum value of the number of function evaluations when the problem is successfully solved. If there is no record of a successful solution, the range of the horizontal axis is from 1000 function evaluations to the product of the maximum number of function evaluations for each problem and the number of problems (reaching this range is regarded as an unsuccessful solution). The vertical axis represents the cumulative number of successfully solved problems. For each record of successful solution, according to the corresponding number of function evaluations, the number of successfully solved problems is accumulated at the corresponding position on the horizontal axis. Finally, the cumulative number of successfully solved problems corresponding to each point is determined through cumulative summation.

**Figure 9:**
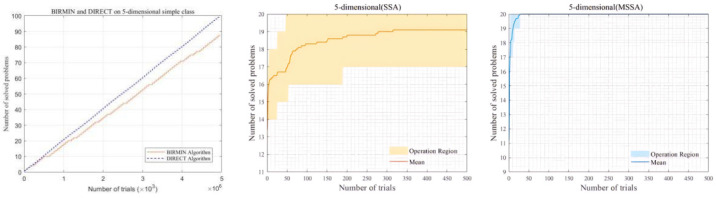
Performance comparison on GKLS functions.

For these 100 GKLS test functions, 20 representative test functions are selected from them. The MSSA algorithm sets the population size to 30 and runs independently 10 times to construct an operation region. During each run, the iteration curve is recorded. This curve can reflect the changes in the optimal solution of the algorithm at different iteration steps. After each run, the error between the final solution and the global optimal value is checked. If the error is less than the tolerance, it is determined that the corresponding problem has been successfully solved in this run. In addition, during each iteration, the number of problems successfully solved by all functions at the current iteration number is also counted. The Figure 9 shows the results of different algorithms solving GKLS test functions.

From the Figure 9, when the two deterministic algorithms, DIRECT and BIRMIN, solve 100 GKLS functions, the number of successfully solved problems gradually increases as the number of function evaluations rises, with DIRECT having a slightly higher efficiency. When the SSA solves 20 GKLS functions, the number of solved problems increases gently. The MSSA, when solving the same 20 GKLS functions, can quickly solve a larger number of problems with fewer trials. Evidently, the MSSA performs outstandingly in global optimization. Although it is a heuristic algorithm, it performs comparably to deterministic algorithms based on rigorous theories, and even has an edge in terms of search efficiency.

## 5. Performance on Engineering Optimization Problems

Addressing real-world engineering problems is a primary goal in the research of swarm intelligence algorithms [46]. Although the results from benchmark test functions provide valuable insights into the performance of an algorithm, they do not fully capture its effectiveness in practical applications. To assess the performance of the improved Sparrow Search Algorithm (MSSA) in engineering tasks, this study investigates three representative engineering problems: the welded beam design problem, the gear reducer design problem, and the cantilever beam design problem. The brief descriptions and graphical references of the three engineering design problems mentioned in the article can be found in reference [47].

In order to evaluate the effectiveness and feasibility of the enhanced MSSA for solving these engineering challenges, five algorithms were selected for comparison: SSA, Chimp, HHO, DBO, and WOA. Each algorithm was independently executed 30 times. The optimal results in the table are displayed in bold italics. The evaluation metrics include the mean, variance, and convergence curves of the experimental results, aiming to derive optimal solutions for each of the engineering problems.

### 5.1. Welded Beam Design Problem

**Figure 10:**
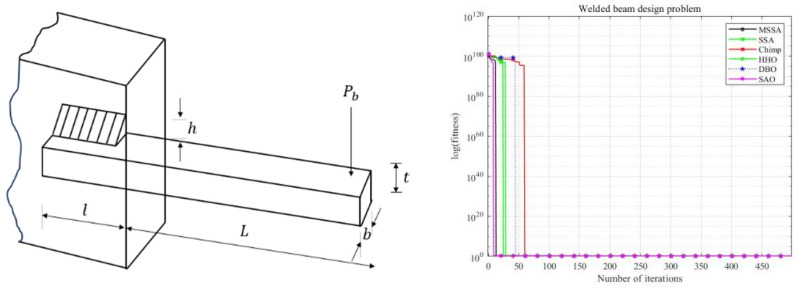
Welded beam design problem’s image and its convergence.

The objective of the welded beam design problem is to minimize manufacturing costs while ensuring safety performance. The problem involves optimizing four variables: weld seam height (h), connection beam length (L), connection beam height (t), and connection beam thickness (b). The image of the welded beam design model and the iteration convergence curve are shown in Figure 10.

From Figure 10 and Table 7, it can be observed that MSSA outperforms the other five algorithms in terms of both mean value and standard deviation, and exhibits faster convergence speed. This observation suggests that MSSA has a competitive advantage in addressing the welded beam design problem.

### 5.2. Speed Reducer Design Problem

**Figure 11:**
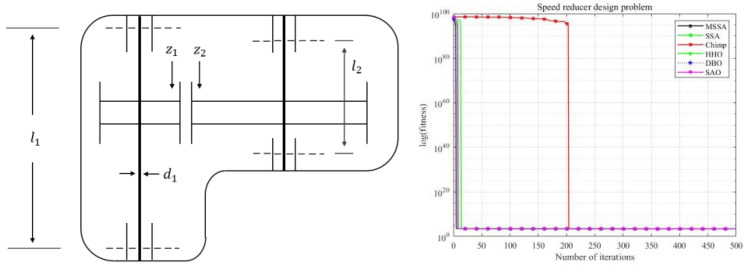
Speed reducer design problem’s image and its convergence.

Speed reducers are indispensable components in mechanical systems, serving as core elements in gearboxes and finding widespread applications across various fields. In this optimization problem, seven variables are to be considered and the objective is to minimize the weight of the gearbox while satisfying 11 constraints. The image of the speed reduction design model and the iteration convergence curve are shown in Figure 11.

From Figure 11 and Table 8, it can be observed that MSSA outperforms the other five algorithms in terms of both mean value and standard deviation, and exhibits faster convergence speed. This suggests that MSSA can provide valuable guidance for determining optimal settings of the seven variables to minimize the weight of the speed reducer.

### 5.3. Cantilever Beam Design Problem

**Figure 12:**
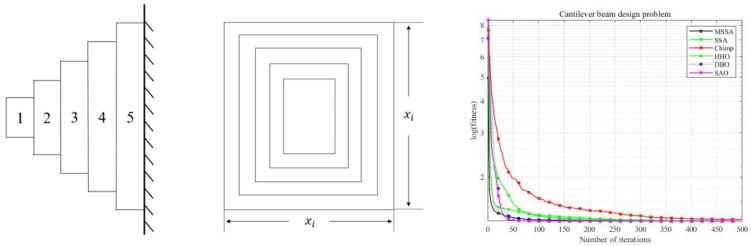
Cantilever beam design problem’s image and its convergence.

The cantilever beam design problem pertains to a structural engineering scenario wherein the primary objective involves the reduction or minimization of the weight associated with the cantilever arm. The cantilever beam arm depicted in the figure is rigidly supported at one end, with vertical forces applied to the free nodes of the cantilever. The beam comprises five hollow cells, each featuring a uniform hollow cross-section with variable height (or width) dimensions, and a constant thickness (here 2/3). The image of the cantilever design model and the iteration convergence curve are shown in Figure 12.

As shown in Figure 12 and Table 9, although the MSSA exhibits lower mean values and standard deviations compared to DBO and SAO, it outperforms SSA and Chimp, while also demonstrating a faster convergence rate. This demonstrates the high effectiveness of utilizing MSSA in addressing the cantilever beam design problem.

## 6. Robot Path Planning Based on MSSA

To evaluate the practicality and feasibility of the modified algorithm, this paper selects a classic robot path planning case for in-depth study [48]. Each sparrow individual is treated as a potential feasible path, assuming there are N possible paths. The dimension of the path is determined by the number of connections between the start and end points. The environment modeling uses a 1 × 1 grid method, transforming the work environment into a plane where obstacles are represented by grid values. A grid value of 0 indicates the feasible domain, while a grid value of 1 indicates the obstacle domain. Therefore, the robot can plan a path within the feasible domain. The dimension represents the number of columns in the grid map, and the path cost function represents the path cost of the i−th sparrow individual, as shown below:

(9) $$f(x_{i})=\sum_{j=1}^{D-1}\sqrt{{\left(x_{j+1}-x_{j}\right)}^{2}+{\left(y_{j+1}-y_{j}\right)}^{2}}$$

In Equation (9), j is the j−th dimension of a sparrow individual.

### 6.1. Experimental Environment Settings

To validate the practicality of the improved algorithm, it is applied to route a 20 × 20 grid map and compared with GA, SSA, MSSA, ASFSSA, and GWO. The population size is set to 40, and the number of iterations is 100, with other parameters kept consistent.

### 6.2. Simulation Results and Analysis

**Figure 13:**
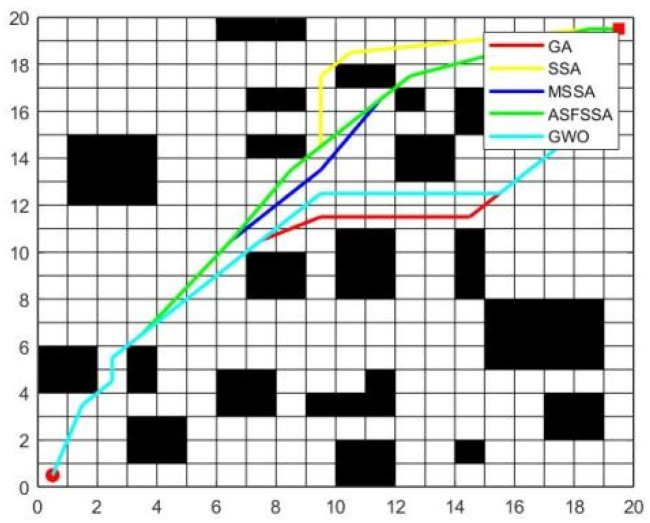
A 20 × 20 shortest path planning diagram.

The optimal route of each algorithm in the 20 × 20 model graphs is shown in Figure 13. To eliminate the influence of randomness, each algorithm is tested 10 times, and the best, average, and worst routes are recorded. Three indicators are used to evaluate the stability and feasibility of each algorithm. The optimization statistical table is shown in Table 10.

From Table 10 and Figure 13, it can be seen that the average shortest path of MSSA is second only to ASFSSA and outperforms GA, SSA, and GWO. Compared to the original SSA, MSSA demonstrates stronger search capability and stability, effectively preventing it from getting trapped in local optima, significantly improving both the algorithm’s performance and stability.

## 7. Conclusions

This paper presents an algorithm named MSSA (Modified Sparrow Search Algorithm), which aims to address the issues of reduced population diversity, weakened search ability, and a tendency to be trapped in local optima during the later iterations of the Sparrow Search Algorithm (SSA). In the initialization phase, the MSSA algorithm incorporates Latin Hypercube Sampling (LHS) to enhance population diversity. It adopts an adaptive weighting mechanism to boost search efficiency and employs Cauchy mutation and cat swarm disturbance strategies during the discovery phase to improve the global search capability. Experimental results demonstrate that the MSSA performs outstandingly in terms of optimization performance and problem solving, showing great potential for practical applications. To evaluate the performance of MSSA comprehensively, tests were carried out on 23 benchmark functions, 10 CEC2019 test functions, and GKLS test functions in five-dimensional and ten-dimensional spaces. Additionally, MSSA was applied to solve three real-world engineering problems and a 20 × 20 robot path-planning problem.

To provide a clearer overview of the contributions of the MSSA algorithm discussed above, please refer to Table 11.

Statistical test indicators (such as mean and standard deviation) show that, compared to seven other algorithms, MSSA demonstrates higher convergence accuracy and better stability on most test functions. Simulation curves further illustrate that the algorithm converges faster and achieves higher precision. The application of MSSA to real-world engineering problems also further validates the improvements made by the algorithm. The results of the non-parametric Wilcoxon signed-rank test and Friedman rank sum test show that MSSA exhibits a significant difference from other algorithms at the 0.05 significance level.

Although the parameters of the MSSA algorithm were set in this study, these parameters may not be optimal in different scenarios. When dealing with problems of different scales and complexity levels, they may need to be readjusted and optimized. Meanwhile, there is a gap between the test functions used to verify the performance of the MSSA algorithm and real-world problems. Therefore, the effectiveness and adaptability of the algorithm in practical scenarios still need to be further verified. In addition, this study assumes that optimization problems have optimal solutions and that the MSSA algorithm can approach the optimal solutions within a reasonable time. However, some complex problems may not have global optimal solutions. This deviation between the assumption and reality may limit the application of the algorithm in extremely complex problems.

Future research will focus on validating the performance of MSSA on additional benchmark functions, such as the CEC2017 and CEC2022 test functions. In addition, the MSSA algorithm will be enhanced by incorporating techniques like elite reverse strategy, fast non-dominated sorting, mutation factor selection, and archive gene pools. These improvements will enable the evolution of MSSA into a multi-objective sparrow search algorithm, making it suitable for solving real-world optimization problems. For instance, in intelligent transportation systems, MSSA can be used to optimize traffic flow management and path planning; in energy management, it can help improve the thermal efficiency of coal-fired boiler units while reducing pollutant emissions. Furthermore, MSSA shows great potential across various fields, including environmental protection, robot path planning, and aerospace. As the demand for efficient optimization algorithms continues to grow in various industries, the multi-objective optimization capability of MSSA will provide more accurate and effective solutions in these domains.

## Figures and Tables

**Table 1:** Several Sampling Methods.

Sampling Methods	Characteristics	Advantages	Disadvantages
Random Sampling	Samples are randomly distributed within the interval.	Easy to implement, suitable for large-scale sampling.	Samples may cluster in some areas, leading to uneven coverage.
Latin Hypercube Sampling (LHS)	Multidimensional stratified	Can effectively cover the entire range even with few samples.	More complex to compute compared to the tails.
Uniform Distribution Sampling	Each point in the interval has the same probability of being selected.	Simple and easy to implement.	Samples may cluster in certain areas, resulting in uneven coverage.
Gaussian Distribution Sampling	Samples are distributed around the mean, with fewer samples far from the mean.	Suitable for normally distributed data, easy to generate.	Samples are concentrated near the mean, with sparse coverage in the tails.

**Table 2:** The complexity and parameters of each algorithm.

Algorithm	Parameters	Population
SCA	α=2	Population = 40
WOA	b=1, α=2→0, linearly decrease	
HHO	$E_{0}\in (-1,1)$	
Chimp	m=chaos(3,1,1)	
SSA	ST=0.7, PD=0.5, SD=0.3	
ASFSSA	ST=0.7, PD=0.5, SD=0.3	
DBO	α=1 or−1, b=0.3, k=0.1, S=0.5, P_percent=0.2	
SAO	$N_{a}=N_{b}=\frac{N}{2}$	
GA	pm=0.8,pc=0.05	
CPO	$r_{1},r_{2}\in (0,1)$	
MSO	α∈(0,1)	

**Table 3:** Experimental results of MSSA and other algorithms on 23 benchmark functions (D = 30).

	Function	Parameters	SSA	DBO	SAO	SCA	Chimp	HHO	WOA	MSSA
Uni-modal functions	F1	Mean	4.8403 × 10^−244^	1.6633 × 10^−97^	3.9791 × 10^−5^	3.026	8.0807 × 10^−7^	1.2148 × 10^−101^	4.5632 × 10^−75^	0
		SD	0	7.4385 × 10^−97^	6.0809 × 10^−5^	5.2634	2.0318 × 10^−6^	4.7343 × 10^−101^	2.0406 × 10^−74^	0
		*p*-values	0.125	8.8575 × 10^−5^	8.8575 × 10^−5^	8.8575 × 10^−5^	8.8575 × 10^−5^	8.8575 × 10^−5^	8.8575 × 10^−5^	NA
	F2	Mean	1.1962 × 10^−7^	2.8036 × 10^−65^	8.8091 × 10^−4^	1.5654 × 10^−2^	7.0217 × 10^−6^	5.5887 × 10^−52^	1.9709 × 10^−53^	1.2942 × 10^−273^
		SD	2.6373 × 10^−7^	1.2459 × 10^−64^	1.3044 × 10^−3^	3.2183 × 10^−2^	8.563 × 10^−6^	2.2355 × 10^−51^	5.9608 × 10^−53^	0
		*p*-values	3.5153 × 10^−4^	8.8575 × 10^−5^	8.8575 × 10^−5^	8.8575 × 10^−5^	8.8575 × 10^−5^	8.8575 × 10^−5^	8.8575 × 10^−5^	NA
	F3	Mean	9.0416 × 10^−268^	2.3775 × 10-^83^	1066.3701	5856.0238	154.2924	1.917 × 10^−83^	33,479.2865	0
		SD	0	1.0602 × 10^−82^	1446.7758	3755.2895	435.3517	8.1173 × 10^−83^	13,578.1384	0
		*p*-values	3.125 × 10^−2^	8.8575 × 10^−5^	8.8575 × 10^−5^	8.8575 × 10^−5^	8.8575 × 10^−5^	8.8575 × 10^−5^	8.8575 × 10^−5^	NA
	F4	Mean	3.3225 × 10^−107^	4.4094 × 10^−52^	1.423	30.1469	0.11623	1.0448 × 10^−50^	47.6442	3.2137 × 10^−267^
		SD	1.4859 × 10^−106^	1.8761 × 10^−51^	0.57081	10.4015	8.5933 × 10^−2^	2.8154 × 10^−50^	31.7755	0
		*p*-values	4.3778 × 10^−4^	8.8575 × 10^−5^	8.8575 × 10^−5^	8.8575 × 10^−5^	8.8575 × 10^−5^	8.8575 × 10^−5^	8.8575 × 10^−5^	NA
	F5	Mean	2.0593 × 10^−5^	25.4035	40.4379	43,449.6197	28.8927	4.184 × 10^−3^	27.8302	7.7089 × 10^−6^
		SD	5.3199 × 10^−5^	0.22367	27.6976	120,561.791	9.5663 × 10^−2^	6.0131 × 10^−3^	0.47304	1.8361 × 10^−5^
		*p*-values	0.16718	8.8575 × 10^−5^	8.8575 × 10^−5^	8.8575 × 10^−5^	8.8575 × 10^−5^	1.0335 × 10^−4^	8.8575 × 10^−5^	NA
	F6	Mean	1.1962 × 10^−7^	4.0449 × 10^−7^	3.3332 × 10^−5^	9.1999	3.1781	8.4771 × 10^−5^	0.16175	5.3953 × 10^−8^
		SD	2.6373 × 10^−7^	5.2474 × 10^−7^	3.0624 × 10^−5^	7.6902	0.44439	1.6742 × 10^−4^	0.1323	1.1408 × 10^−7^
		*p*-values	0.64416	6.8061 × 10^−4^	8.8575 × 10^−5^	8.8575 × 10^−5^	8.8575 × 10^−5^	8.8575 × 10^−5^	8.8575 × 10^−5^	NA
	F7	Mean	2.741 × 10^−4^	1.8578 × 10^−3^	0.4284	7.5726 × 10^−2^	2.0733 × 10^−3^	1.3783 × 10^−4^	1.9485 × 10-3	1.1603 × 10^−4^
		SD	2.493 × 10^−4^	1.2634 × 10^−3^	1.7508 × 10^−2^	7.8847 × 10^−2^	1.8934 × 10^−3^	1.6957 × 10^−4^	1.6768 × 10^−3^	8.5419 × 10^−5^
		*p*-values	4.7858 × 10^−2^	8.8575 × 10^−5^	8.8575 × 10^−5^	8.8575 × 10^−5^	8.8575 × 10^−5^	0.88129	1.6286 × 10^−4^	NA
Multi-modal functions	F8	Mean	−9502.1132	−8870.9105	−9120.8181	−3768.5139	−5730.4026	−12,569.1605	−10,973.321	−10,879.7721
		SD	2738.0434	1446.006	772.1644	243.0804	62.194	0.54721	2094.2708	1794.4138
		*p*-values	0.21796	5.734 × 10^−3^	2.495 × 10^−3^	8.8575 × 10^−5^	1.0335 × 10^−4^	8.8575 × 10^−5^	0.68132	NA
	F9	Mean	0	2.4898	38.1862	36.0641	9.075	0	0	0
		SD	0	6.8064	15.5384	38.0386	7.8769	0	0	0
		*p*-values	1	0.125	8.8575 × 10^−5^	8.8575 × 10^−5^	8.8575 × 10^−5^	1	1	NA
	F10	Mean	4.4409 × 10^−16^	4.4409 × 10^−16^	1.4674 × 10^−3^	16.5063	19.9622	4.4409 × 10^−16^	2.7534 × 10^−15^	4.4409 × 10^−16^
		SD	0	0	1.459 × 10^−3^	7.6507	1.3489 × 10^−3^	0	1.7386 × 10^−15^	4.4409 × 10^−16^
		*p*-values	1	1	8.8575 × 10^−5^	8.8575 × 10^−5^	8.8575 × 10^−5^	1	2.4414 × 10^−4^	NA
	F11	Mean	0	0	5.2997 × 10^−2^	0.7005	2.5468 × 10^−2^	0	4.2266 × 10^−3^	0
		SD	0	0	2.0365 × 10^−1^	0.31924	4.3681 × 10^−2^	0	1.8902 ×	0
		*p*-values	1	1	8.8575 × 10^−5^	8.8575 × 10^−5^	8.8575 × 10^−5^	1	1	NA
	F12	Mean	3.6802 × 10^−8^	2.6807 × 10^−4^	1.0371 × 10^−2^	45,201.469	0.3697	2.4008 × 10^−6^	1.3899 × 10^−2^	1.2762 × 10^−8^
		SD	6.6565 × 10-8	1.1283 × 10^−3^	3.1908 × 10^−2^	202,001.79	0.14812	2.5829 × 10^−6^	1.0749 × 10^−2^	1.5159 × 10^−8^
		*p*-values	0.29588	5.6915 × 10^−2^	8.8575 × 10^−5^	8.8575 × 10^−5^	8.857 × 10^−5^	8.8575 × 10^−5^	8.8575 × 10^−5^	NA
	F13	Mean	4.1787 × 10^−7^	6.527 × 10^−2^	4.4476 × 10^−3^	60,807.8872	2.7933	8.5529 × 10^−5^	0.32628	2.1011 × 10^−7^
		SD	6.2505 × 10^−7^	0.11389	0.10309	164,292.136	0.12083	6.9127 × 10^−5^	0.1877	4.1398 × 10^−7^
		*p*-values	3.6561 × 10^−2^	8.8575 × 10^−5^	1.4013 × 10^−4^	8.8575 × 10-5	8.8575 × 10^−5^	1.0335 × 10^−4^	8.8575 × 10^−5^	NA
Fixed-dimensional multi-modal functions	F14	Mean	7.1949	1.1964	3.0155	1.891	0.99812	1.4931	2.6614	0.998
		SD	5.0208	0.61069	3.0155	1.0126	4.3042 × 10^−4^	1.1321	3.5292	2.3447 × 10^−9^
		*p*-values	1.1529 × 10^−4^	0.72656	4.8828 × 10^−4^	8.8575 × 10^−5^	8.8575 × 10^−5^	6.8061 × 10^−4^	5.9342 × 10^−4^	NA
	F15	Mean	3.1787 × 10^−4^	8.648 × 10^−4^	2.5917 × 10^−3^	1.0903 × 10^−3^	1.2823 × 10^−3^	3.762 × 10^−4^	5.7833 × 10^−4^	3.5493 × 10^−4^
		SD	2.0142 × 10^−5^	3.2621 × 10^−4^	6.0923 × 10^−3^	4.0239 × 10^−4^	3.7148 × 10^−5^	2.072 × 10^−4^	2.8029 × 10^−4^	2.0437 × 10^−4^
		*p*-values	0.2988	3.3385 × 10^−4^	2.2039 × 10^−3^	8.8575 × 10^−5^	8.8575 × 10^−5^	2.2821 × 10^−3^	1.3245 × 10^−3^	NA
	F16	Mean	−1.0316	−1.0316	−1.0316	−1.0316	−1.0316	−1.0316	−1.0316	−1.0316
		SD	1.4408 × 10^−16^	2.0376 × 10^−16^	2.2781 × 10^−16^	2.9831 × 10^−5^	9.7388 × 10^−6^	2.0397 × 10^−10^	1.3452 × 10^−10^	1.3478 × 10^−16^
		*p*-values	1	1	1	8.8575 × 10^−5^	8.8575 × 10^−5^	1.1964 × 10^−4^	8.8575 × 10^−5^	NA
	F17	Mean	0.39789	0.39789	0.39789	0.39958	0.39898	0.39789	0.39789	0.39789
		SD	0	0	0	1.1511 × 10^−3^	1.4041 × 10^−3^	9.9056 × 10^−6^	1.4366 × 10^−5^	1.1917 × 10^−15^
		*p*-values	1	1	1	8.8575 × 10^−5^	8.8575 × 10^−5^	1.9644 × 10^−4^	8.8575 × 10^−5^	NA
	F18	Mean	3	3	3	3	3.0001	3	3	3
		SD	2.849 × 10^−15^	1.5214 × 10^−15^	4.5563 × 10^−16^	1.5395 × 10^−5^	1.3295 × 10^−4^	1.9892 × 10^−7^	6.0152 × 10^−5^	1.1246 × 10^−13^
		*p*-values	1.1985 × 10^−4^	1.2257 × 10^−4^	7.9305 × 10^−5^	8.8575 × 10^−5^	8.8575 × 10^−5^	1.0178 × 10^−3^	8.8575 × 10^−5^	NA
	F19	Mean	−3.8628	−3.8616	−3.8628	−3.8553	−3.8552	−3.8611	−3.8579	−3.8628
		SD	3.3348 × 10^−14^	2.8874 × 10^−3^	2.2781 × 10^−15^	3.3086 × 10^−3^	2.1766 × 10^−3^	3.9838 × 10^−3^	3.9586 × 10^−3^	1.278 × 10^−9^
		*p*-values	8.8575 × 10^−5^	7.3138 × 10^−3^	8.8575 × 10^−5^	8.8575 × 10^−5^	8.8575 × 10^−5^	8.8575 × 10^−5^	8.8575 × 10^−5^	NA
	F20	Mean	−3.2739	−3.2076	−3.2685	−2.9212	−2.5851	−3.1395	−3.1855	−3.2654
		SD	6.7875 × 10^−2^	0.11195	6.0685 × 10^−2^	0.20153	0.4862	9.1708 × 10^−2^	0.20373	8.0715 × 10^−2^
		*p*-values	0.70891	0.21796	0.37026	1.4013 × 10^−4^	8.8575 × 10^−5^	1.1713 × 10^−3^	0.20433	NA
	F21	Mean	−10.1532	−7.0682	−5.4464	−3.3795	−3.5228	−5.0536	−7.8775	−10.1532
		SD	8.5674 × 10^−8^	2.9472	1.6944	1.8885	2.0319	1.5366 × 10^−3^	3.2513	2.3441 × 10^−13^
		*p*-values	0.17212	7.7877 × 10^−4^	1.4599 × 10^−4^	8.8575 × 10^−5^	8.8575 × 10^−5^	8.8575 × 10^−5^	8.8575 × 10^−5^	NA
	F22	Mean	−10.4029	−7.9039	−6.4516	−1.6658	−4.1684	−5.6137	−8.5163	−10.4029
		SD	2.0709 × 10^−7^	2.8795	2.76	1.5221	1.6727	1.6262	2.8964	1.2333 × 10^−11^
		*p*-values	0.70467	1.2264 × 10^−2^	1.2673 × 10^−3^	8.8575 × 10^−5^	8.8575 × 10^−5^	8.8575 × 10^−5^	8.8575 × 10^−5^	NA
	F23	Mean	−10.266	−8.4347	−6.9566	−4.3889	−4.824	−5.393	7.	−10.5364
		SD	1.2092	2.9169	2.7101	1.8821	0.91442	1.1914	3.3698	1.1691 × 10^−10^
		*p*-values	0.55658	2.1682 × 10^−2^	4.0324 × 10^−3^	8.8575 × 10^−5^	8.8575 × 10^−5^	8.8575 × 10^−5^	8.8575 × 10^−5^	NA

Note: NA in the table indicates invalid data.

**Table 4:** Experimental results of MSSA and other algorithms on F1–F13 functions.

Function	Algorithms	D=50		D=100	
		Mean	SD	Mean	SD
F1	MSSA	0	0	0	0
	SSA	1.3087 × 10^−235^	0	9.1969 × 10^−289^	0
	ASFSSA	0	0	0	0
F2	MSSA	0	0	1.6393 × 10^−282^	0
	SSA	2.0532 × 10^−6^	9.1280 × 10^−86^	4.8594 × 10^−139^	2.1732 × 10^−138^
	ASFSSA	3.2924 × 10^−311^	0	3.1895 × 10^−280^	0
F3	MSSA	0	0	0	0
	SSA	1.4521 × 10^−210^	0	4.8765 × 10^−130^	2.1808 × 10^−129^
	ASFSSA	0	0	0	0
F4	MSSA	5.990 × 10^−249^	0	0	0
	SSA	1.4296 × 10^−127^	6.3935 × 10^−127^	2.2598 × 10^−126^	1.0106 × 10^−125^
	ASFSSA	2.7552 × 10^−304^	0	3.5802 × 10^−255^	0
F5	MSSA	5.2803 × 10^−5^	1.0789 × 10^−4^	2.0216 × 10^−4^	3.0459 × 10^−4^
	SSA	7.8868 × 10^−5^	1.1923 × 10^−4^	4.1506 × 10^−4^	9.9227 × 10^−4^
	ASFSSA	4.9156 × 10^−3^	8.6149 × 10^−3^	1.6051 × 10^−2^	3.459 × 10^−2^
F6	MSSA	2.3840 × 10^−7^	3.5863 × 10^−7^	6.6802 × 10^−7^	8.8676 × 10^−7^
	SSA	4.3318 × 10^−7^	5.8853 × 10^−7^	1.5188 × 10^−6^	2.3585 × 10^−6^
	ASFSSA	1.2079 × 10^−5^	2.5184 × 10^−5^	9.1211 × 10^−5^	1.9119 × 10^−4^
F7	MSSA	1.3568 × 10^−4^	1.2107 × 10^−4^	1.2734 × 10^−4^	1.0386 × 10^−4^
	SSA	2.5163 × 10^−4^	1.2289 × 10^−4^	2.4166 × 10^−4^	1.62621 × 10^−4^
	ASFSSA	9.8284 × 10^−5^	1.2425 × 10^−4^	1.0428 × 10^−4^	1.0206 × 10^−4^
F8	MSSA	−17,708.5754	2721.5471	−36,630.683	3971.7559
	SSA	−17,278.536	3586.9176	−37,485.9043	4113.869
	ASFSSA	−15,546.3062	1071.4226	−22,423.7605	2050.4667
F9	MSSA	0	0	0	0
	SSA	0	0	0	0
	ASFSSA	0	0	0	0
F10	MSSA	4.4409 × 10^−16^	0	4.4409 × 10^−16^	0
	SSA	4.4409 × 10^−16^	0	4.4409 × 10^−16^	0
	ASFSSA	4.4409 × 10^−16^	0	4.4409 × 10^−16^	0
F11	MSSA	0	0	0	0
	SSA	0	0	0	0
	ASFSSA	0	0	0	0
F12	MSSA	1.8017 × 10^−8^	4.0916 × 10^−8^	2.4191 × 10^−8^	7.6385 × 10^−8^
	SSA	3.9135 × 10^−9^	7.3722 × 10^−9^	2.9194 × 10^−8^	6.2248 × 10^−8^
	ASFSSA	1.9148 × 10^−7^	2.8972 × 10^−7^	2.3519 × 10^−7^	3.7433 × 10^−7^
F13	MSSA	4.3383 × 10^−7^	5.6604 × 10^−7^	1.0375 × 10^−6^	2.2243 × 10^−6^
	SSA	9.1585 × 10^−7^	2.6195 × 10^−6^	2.1674 × 10^−6^	3.3805 × 10^−6^
	ASFSSA	5.0759 × 10^−6^	7.0915 × 10^−6^	1.9273 × 10^−5^	2.9267 × 10^−5^

**Table 5:** Experimental results of MSSA and other algorithms on F1–F13 functions (D = 30).

Function	Parameters	MSSA	CPO	CSSA	MSO
F1	Mean	0	1.3957 × 10^−148^	0	1.032 × 10^−3^
	SD	0	3.797 × 10^−148^	0	8.1508 × 10^−4^
	*p*-values	NA	1.9531 × 10^−3^	1	1.9531 × 10^−3^
F2	Mean	0	2.8512 × 10^−66^	2.9768 × 10^−200^	5.3912 × 10^−3^
	SD	0	9.0161 × 10^−66^	0	4.4073 × 10^−3^
	*p*-values	NA	1.9531 × 10^−3^	0.5	1.9531 × 10^−3^
F3	Mean	0	3.4518 × 10^−121^	0	1952.2359
	SD	0	1.0915 × 10^−120^	0	755.6469
	*p*-values	NA	1.9531 × 10^−3^	1	1.9531 × 10^−3^
F4	Mean	1.538 × 10^−320^	2.0157 × 10^−63^	5.164 × 10^−63^	5.164 × 10^−225^
	SD	0	4.7631 × 10^−63^	0	5.5849
	*p*-values	NA	1.9531 × 10^−3^	0.5	1.9531 × 10^−3^
F5	Mean	5.5967 × 10^−6^	24.9892	1.5787 × 10^−4^	214.0628
	SD	8.888 × 10^−6^	8.7853	2.0579 × 10^−4^	301.0648
	*p*-values	NA	1.9531 × 10^−3^	9.7656 × 10^−3^	1.9531 × 10^−3^
F6	Mean	2.5788 × 10^−8^	6.1026 × 10^−2^	1.1519 × 10^−6^	9.5927 × 10^−4^
	SD	4.4412 × 10^−8^	1.1005 × 10^−1^	1.3663 × 10^−6^	4.1921 × 10^−4^
	*p*-values	NA	1.9531 × 10^−3^	3.9062 × 10^−3^	1.9531 × 10^−3^
F7	Mean	1.252 × 10^−4^	1.115 × 10^−4^	1.5085 × 10^−4^	9.1615 × 10^−2^
	SD	1.4052 × 10^−4^	9.9963 × 10^−5^	1.0329 × 10^−4^	3.3619 × 10^−2^
	*p*-values	NA	0.76953	0.49219	1.9531 × 10^−3^
F8	Mean	−10,511.7565	−4353.8839	−12,200.0677	−9583.8378
	SD	1722.7594	1933.8196	563.9687	328.9952
	*p*-values	NA	1.9531 × 10^−3^	3.9062 × 10^−3^	1.6016 × 10^−1^
F9	Mean	0	0	0	36.6183
	SD	0	0	0	11.571
	*p*-values	NA	1	1	1.9531 × 10^−3^
F10	Mean	4.4409 × 10^−16^	4.4409 × 10^−16^	4.4409 × 10^−16^	4.6258 × 10^−1^
	SD	0	0	0	6.1442 × 10^−1^
	*p*-values	NA	1	1	1.9531 × 10^−3^
F11	Mean	0	0	0	2.1175 × 10^−2^
	SD	0	0	0	1.8055 × 10^−2^
	*p*-values	NA	1	1	1.9531 × 10^−3^
F12	Mean	1.2615 × 10^−8^	3.6508 × 10^−7^	5.2542 × 10^−8^	3.2297 × 10^−1^
	SD	2.0312 × 10^−8^	3.5871 × 10^−7^	7.4516 × 10^−8^	4.3165 × 10^−1^
	*p*-values	NA	1.9531 × 10^−3^	2.3242 × 10^−3^	1.9532 × 10^−3^
F13	Mean	1.9609 × 10^−7^	3.9974 × 10^−6^	1.5651 × 10^−6^	2.5870 × 10^−1^
	SD	2.7869 × 10^−7^	4.2964 × 10^−6^	2.2656 × 10^−6^	7.9157 × 10^−1^
	*p*-values	NA	9.7656 × 10^−3^	2.3242 × 10^−1^	1.9531 × 10^−3^

Note: NA in the table indicates invalid data.

**Table 6:** Experimental results of MSSA and other algorithms on CEC2019.

Function	Parameters	MSSA	SSA	Chimp	HHO	DBO	SAO
F1	Mean	1.0000	1.0000	1,781,542.16	1.0000	821,233.12	17,947.94
	SD	0.0000	0.0000	3,096,970.03	0.0000	3,264,743.61	23,363.7653
F2	Mean	4.9748	5.0000	2307.6197	5.0000	457.5043	166.2708
	SD	0.1380	0.0000	1300.2847	0.0000	1201.2186	101.6676
F3	Mean	4.4510	6.4640	5.6428	4.5391	4.7095	2.7336
	SD	2.3754	2.7549	1.1436	0.9620	2.3807	2.0580
F4	Mean	1.0000	1.0000	1.0000	1.0000	1.0000	1.0000
	SD	0.0000	0.0000	0.0000	0.0000	0.0000	0.0000
F5	Mean	1.0000	1.0000	1.0000	1.0000	1.0000	1.0000
	SD	0.0000	0.0000	0.0000	0.0000	0.0000	0.0000
F6	Mean	4.1031	4.2008	4.6667	4.2685	4.4228	4.1928
	SD	0.3198	0.4407	0.3638	0.3985	0.3279	0.3166
F7	Mean	1.0000	1.0000	1.0028	1.0000	1.0000	1.0042
	SD	0.0000	0.0000	0.0107	0.0000	0.0000	0.0158
F8	Mean	1.0000	1.0000	1.0000	1.0000	1.0000	1.0000
	SD	0.0000	0.0000	0.0000	0.0000	0.0000	0.0000
F9	Mean	1.0000	1.0000	1.0000	1.0000	1.0000	1.0000
	SD	0.0000	0.0000	0.0000	0.0000	0.0002	0.0000
F10	Mean	1.0000	1.0000	1.0000	1.0000	1.0000	1.0000
	SD	0.0000	0.0000	0.0000	0.0000	0.0000	0.0000
Friedman Score		3.0049	3.4633	4.5883	3.2800	3.5500	3.5480
Friedman Rank		1	3	6	2	5	4

**Table 7:** Comparison of optimization designs for welded beam design using different algorithms.

Algorithm	Mean	SD
MSSA	1.7187	0.0586
SSA	1.9794	0.3861
Chimp	1.7969	0.0268
HHO	2.0326	0.3202
DBO	1.7329	0.0843
SAO	1.7154	0.0692

**Table 8:** Comparison of optimization designs of the speed reducer using different algorithms.

Algorithm	Mean	SD
MSSA	2727.7338	22.0685
SSA	3087.6111	337.1181
Chimp	3130.4727	42.3819
HHO	3051.3012	62.0078
DBO	3027.4794	48.4128
SAO	2994.4711	0.0000

**Table 9:** Comparison of the optimization designs for the cantilever beam using different algorithms.

Algorithm	Mean	SD
MSSA	1.3409	0.0006
SSA	1.3423	0.0018
Chimp	1.3628	0.0091
HHO	1.3431	0.0021
DBO	1.3400	0.0000
SAO	1.3400	0.0000

**Table 10:** Statistics table of optimization route by algorithms.

Metrics	GA	SSA	MSSA	ASFSSA	GWO
Best	28.0192	28.5777	28.5777	28.4193	28.4193
Mean	29.3141	29.9852	28.8836	28.5875	29.3287
Worse	30.4869	31.1395	29.7765	28.6315	30.8721

**Table 11:** Key contributions and performance validation of the MSSA algorithm.

Contribution	Description
Enhancement of Population Diversity	Introduced Latin Hypercube Sampling (LHS) during the initialization phase to enhance population diversity and avoid premature convergence.
Adaptive Weighting Mechanism	Applied an adaptive weighting mechanism to improve search efficiency, ensuring optimal performance at different stages of the search process.
Enhanced Global Search Capability	Utilized Cauchy mutation and cat disturbance strategies during the discovery phase to strengthen global search ability and prevent premature convergence to local optima.
Optimization Performance Validation	To verify the optimization performance and global optimization ability of the Modified Sparrow Search Algorithm (MSSA), tests were conducted on 23 benchmark functions, 10 CEC2019 test functions, and 100 five-dimensional GKLS test functions.
Stability and Precision	Experimental results indicate that MSSA outperforms other algorithms in terms of convergence precision and stability on most test functions.
Application to Real-World Problems	Demonstrated the effectiveness of MSSA by applying it to three real-world engineering problems, and a 20 × 20 robot path-planning problem further validating the improvements made.
Statistical Tests	Wilcoxon signed-rank test showed significant differences between MSSA and other algorithms at a 0.05 significance level.

## Data Availability

This manuscript does not report data generation or analysis.

## References

[1] Yun L. (2011). MATLAB implementation of Newton’s iteration method. Inf. Commun..

[2] Ruder S. (2016). An overview of gradient descent optimization algorithms. arXiv.

[3] Cao L., Cai Y., Yue Y. (2019). Swarm Intelligence-Based Performance Optimization for Mobile Wireless Sensor Networks: Survey, Challenges, and Future Directions. IEEE Access.

[4] Zhang J.J., Li J.D., Xu X.M. (2023). A Passive Positioning Algorithm Using Time-Frequency Differences Based on the Cuckoo Search Algorithm and the Newton Method. Electron. Des. Eng..

[5] Izuchukwu C., Shehu Y. (2023). A new inertial projected reflected gradient method with application to optimal control problems. Optim. Methods Softw..

[6] Sakovich N., Aksenov D., Pleshakova E., Gataullin S. (2024). MAMGD: Gradient-Based Optimization Method Using Exponential Decay. Technologies.

[7] Yan F., Xu Y. An Optimized MTD Moving Target Detection Algorithm Based on Gradient Descent with Sampling Point Weights. Proceedings of the 22nd Academic Annual Conference on Vacuum Electronics.

[8] Ye R.Z., Du F.Z. (2024). A Multi-Objective Fuzzy Optimization Scheduling Method for Regional Power Grids Based on the Distributed Newton Method. Electr. Technol. Econ..

[9] Kennedy J. Particle Swarm Optimization. Proceedings of the 1995 IEEE International Conference on Neural Networks.

[10] Han Y., Cai J., Zhou G., Li Y., Lin H., Tang J. (2010). Research Progress of Random Frog Leaping Algorithm. Comput. Sci..

[11] Qin Q., Cheng S., Li L., Shi Y. (2014). Artificial Bee Colony Algorithm: A Survey. Appl. Math. Comput..

[12] Mirjalili S., Mirjalili S.M., Lewis A. (2014). Grey Wolf Optimizer. Adv. Eng. Softw..

[13] Mirjalili S. (2016). SCA: A Sine Cosine Algorithm for Solving Optimization Problems. Knowl.-Based Syst..

[14] Mirjalili S., Lewis A. (2016). The Whale Optimization Algorithm. Adv. Eng. Softw..

[15] Heidari A.A., Mirjalili S., Faris H., Aljarah I., Mafarja M., Chen H. (2019). Harrishawks Optimization: Algorithm and Applications. Fut. Gener. Comput. Syst..

[16] Khishe M., Mosavi M.R. (2020). Chimp Optimization Algorithm. Expert Syst. Appl..

[17] Xue J., Shen B. (2020). A novel swarm intelligence optimization approach: Sparrow search algorithm. Syst. Sci. Control. Eng..

[18] Xue J., Shen B. (2022). Dung beetle optimizer: A new meta-heuristic algorithm for global optimization. J. Supercomput..

[19] Deng L., Liu S. (2023). Snow Ablation Optimizer: A Novel Metaheuristic Technique for Numerical Optimization and Engineering Design. Expert. Syst. Appl..

[20] Guo Z., Liu G., Jiang F. (2025). Chinese Pangolin Optimizer: A novel bio-inspired metaheuristic for solving optimization problems. J. Supercomput..

[21] He J., Zhao S., Ding J., Wang Y. (2025). Mirage search optimization: Application to path planning and engineering design problems. Adv. Eng. Softw..

[22] Elsisi M. (2024). Optimal Design of Adaptive Model Predictive Control Based on Improved GWO for Autonomous Vehicle Considering System Vision Uncertainty. Appl. Soft Comput..

[23] Chen M., Cheng Q., Feng X., Zhao K., Zhou Y., Xing B., Tang S., Wang R., Duan J., Wang J. (2024). Optimized variational mode decomposition algorithm based on adaptive thresholding method and improved whale optimization algorithm for denoising magnetocardiography signal. Biomed. Signal Process. Control..

[24] Liu H., Fan J., Guo P. (2024). Improved gorilla optimization algorithm for kernel fuzzy clustering segmentation of RGB-D images. Microelectron. Comput..

[25] Javaheri D., Gorgin S., Lee J.A., Masdari M. (2021). An improved discrete Harris hawk optimization algorithm for efficient workflow scheduling in multi-fog computing. Expert. Syst. Appl..

[26] Zhang C., Ma H., Hua L., Sun W., Nazir M.S., Peng T. (2022). An evolutionary deep learning model based on TVFEMD, improved sine cosine algorithm, CNN and BiLSTM for wind speed prediction. Renew. Energy.

[27] Wu D., Yuan C. (2022). Correction to: Threshold image segmentation based on improved sparrow search algorithm. Multimed. Tools Appl..

[28] Panimalar K., Kanmani S. (2022). Energy Efficient Cluster Head Selection Using Improved Sparrow Search Algorithm in Wireless Sensor Networks. J. King Saud Univ. Comput. Inf. Sci..

[29] Fei L., Li R., Liu S.Q., Tang B., Li S., Masoud M. (2022). An Improved Sparrow Search Algorithm for Solving the Energy-Saving Flexible Job Shop Scheduling Problem. Machines.

[30] Zhou N., Zhang S., Zhang C. (2022). Multi Strategy Improved Sparrow Search Algorithm Based on Rough Data Reasoning. J. Univ. Electron. Sci. Technol. China.

[31] Zhang W., Liu S. (2022). Adaptive t-Distribution and Improved Golden Sine Sparrow Search Algorithm and Its Applications. Microelectron. Comput..

[32] Ouyang C., Qiu Y., Zhu D. (2021). Adaptive Spiral Flying Sparrow Search Algorithm. Sci. Prog..

[33] Duan Y., Liu C. (2022). Sparrow Search Algorithm Based on Sobol Sequence and Crisscross Strategy. J. Comput. Appl..

[34] Zhang C., Ding S. (2021). A Stochastic Configuration Network Based on Chaotic Sparrow Search Algorithm. Knowl. Based Syst..

[35] Zhu Y., Yousefi N. (2021). Optimal Parameter Identification of PEMFC Stacks Using Adaptive Sparrow Search Algorithm. Microelectron. Comput..

[36] Liu G., Shu C., Liang Z., Peng B., Cheng L. (2021). A Modified Sparrow Search Algorithm with Application in 3D Route Planning for UAV. Sensors.

[37] Song X., Wu Q., Cai Y. Short-Term Power Load Forecasting Based on GRU Neural Network Optimized by an Improved Sparrow Search Algorithm. Proceedings of the Eighth International Symposium on Advances in Electrical, Electronics, and Computer Engineering (ISAEECE 2023).

[38] Wolpert D.H., Macready W.G. (1997). No Free Lunch Theorems for Optimization. IEEE Trans. Evol. Comput..

[39] Stein M. (1987). Large Sample Properties of Simulations Using Latin Hypercube Sampling. Technometrics.

[40] Lü L., Ji W. (2017). A Particle Swarm Optimization Algorithm Combining Centroid Concept and Cauchy Mutation Strategy. Comput. Appl..

[41] Han R., Zhang X.F. (2016). Pseudo-random sequence generation method based on high-dimensional cat mapping. Comput. Eng. Appl..

[42] Suganthan P.N., Hansen N., Liang J.J., Deb K., Chen Y.-P., Auger A., Tiwari S. (2005). Problem Definitions and Evaluation Criteria for the CEC 2005 Special Session on Real-Parameter Optimization. Nat. Comput..

[43] Price K.V., Awad N.H., Ali M.Z., Suganthan P.N. (2018). Problem Definitions and Evaluation Criteria for the 100-Digit Challenge Special Session and Competition on Single Objective Numerical Optimization.

[44] Gaviano M., Kvasov D.E., Lera D., Sergeyev Y.D. (2003). Algorithm 829: Software for generation of classes of test functions with known local and global minima for global optimization. ACM Trans. Math. Softw..

[45] Sergeyev Y.D., Kvasov D.E., Mukhametzhanov M.S. (2018). On the efficiency of nature-inspired metaheuristics in expensive global optimization with limited budget. Sci. Rep..

[46] Rather S.A., Bala P.S. (2020). Swarm-Based Chaotic Gravitational Search Algorithm for Solving Mechanical Engineering Design Problems. World J. Eng..

[47] Chen P., Zhou S., Zhang Q., Kasabov N. (2022). A Meta-Inspired Termite Queen Algorithm for Global Optimization and Engineering Design Problems. Eng. Appl. Artif. Intell..

[48] Zhao Z.H., Ma J.D., Zhang Y.R. (2024). Research on Robot Path Planning Based on an Improved Particle Swarm Dung Beetle Algorithm. China New Technol. Prod..

